# Notification of changes in taxonomic opinion previously published outside the IJSEM. List of Changes in Taxonomic Opinion no. 42

**DOI:** 10.1099/ijsem.0.006793

**Published:** 2025-07-31

**Authors:** Aharon Oren, Markus Göker

**Affiliations:** 1 The Institute of Life Sciences, The Hebrew University of Jerusalem, The Edmond J. Safra Campus, 9190401 Jerusalem, Israel; 2 Leibniz Institute DSMZ – German Collection of Microorganisms and Cell Cultures, Inhoffenstrasse 7B, 38124 Braunschweig, Germany

The International Code of Nomenclature of Prokaryotes (ICNP) [1] deals with the nomenclature of prokaryotes. This may include existing names (the Approved Lists of Bacterial Names), as well as new names and new combinations. In this sense, the Code is also indirectly dealing with taxonomic opinions. However, as with most codes of nomenclature, there are no mechanisms for formally recording taxonomic opinions that do not involve the creation of new names or new combinations. In particular, it would be desirable for taxonomic opinions resulting from the creation of synonyms or emended descriptions to be made widely available to the public. In 2004, the Editorial Board of the *International Journal of Systematic and Evolutionary Microbiology* (*IJSEM*) agreed unanimously that it was desirable to cover such changes in taxonomic opinions (i.e. the creation of synonyms or the emendation of circumscriptions) previously published outside the *IJSEM* and to introduce a List of Changes in Taxonomic Opinion [2]. Scientists wishing to have changes in taxonomic opinion included in future lists should send a PDF file of the relevant publication to the *IJSEM* Editorial Office or to the Lists Editors.

It must be stressed that the date of proposed taxonomic changes is the date of the original publication, not the date of the publication of the list. Taxonomic opinions included in the List of Changes in Taxonomic Opinion do not affect the status of a name under the ICNP [2] and can be considered neither as approved nor as disapproved by the International Committee on Systematics of Prokaryotes. The names that are to be used are those that are the ‘correct names’ (in the sense of Principle 6 of the ICNP) in the opinion of the microbiologist, with a given circumscription, position and rank. A particular name, circumscription, position and rank do not have to be adopted in all circumstances. Consequently, the List of Changes in Taxonomic Opinion must be considered as a service to microbiology, and it has no ‘official character’, other than providing a centralized point for registering/indexing such changes in a way that makes them easily accessible to the scientific community.

The List Editors reserve the right not to include in the list proposed emendations that are in our opinion unimportant and do not significantly alter the original description of the taxon. This includes the addition of new members to the taxon, reclassification of members in a different taxon, addition of genome sequence data and other changes that marginally change the taxon circumscription [3]. Conversely, the mere inclusion of an emendation in the List of Changes in Taxonomic Opinion does not indicate that the List Editors regard this emendation as taxonomically relevant.

**Table:** 

Name/authors	Proposed as	Reference
*Albidovulum* Albuquerque *et al*. 2003 emend. He *et al*. 2025, 10*	Emend.	[4]
*Alteromonadaceae* Ivanova and Mikhailov 2001 emend. Flores-Félix *et al*. 2025, 10*	Emend.	[5]
*Anoxybacillus geothermalis* Filippidou *et al*. 2016 pro synon. *Anoxybacillus rupiensis* Derekova *et al*. 200*8*	Synon.	[6]
*Anoxybacillus rupiensis* Derekova *et al*. 2007 emend. Inan Bektas *et al*. 2024, 7*	Emend.	[6]
*Aurantiacibacter rhizosphaerae* Lee and Kim 2020 emend. Kim *et al*. 2025, 7*	Emend.	[7]
*Aurantiacibacter zhengii* (Fang *et al*. 2019) Xu *et al*. 2020 emend. Kim *et al*. 2025, 7*	Emend.	[7]
*Bifidobacteriaceae* Stackebrandt *et al*. 1997 emend. Silva *et al*. 2025, 11*	Emend.	[8]
*Celerinatantimonadaceae* Cramer *et al*. 2011 emend. Flores-Félix *et al*. 2025, 10*	Emend.	[5]
*Congregibacter* Spring *et al*. 2009 emend. Tak *et al*. 2024, 745	Emend.	[9]
*Curtobacterium citreum* (Komagata and Iizuka 1964) Yamada and Komagata 1972 (Approved Lists 1980) emend. Osdaghi *et al*. 2024, 10*	Emend.	[10]
*Curtobacterium citreum* (Komagata and Iizuka 1964) Yamada and Komagata 1972 (Approved Lists 1980) pro synon. *Curtobacterium albidum* (Komagata and Iizuka 1964) Yamada and Komagata 1972 (Approved Lists 1980)	Synon.	[10]
*Curtobacterium flaccumfaciens* (Hedges 1922) Collins and Jones 1984 emend. Osdaghi *et al*. 2024, 10*	Emend.	[10]
*Endozoicomonadaceae* Bartz *et al*. 2018 emend. Flores-Félix e*t al*. 2025, 10*	Emend.	[5]
*Exiguobacterium antarcticum* Frühling *et al*. 2002 emend. Wang *et al*. 2024, 5*	Emend.	[11]
*Exiguobacterium artemiae* López-Cortés *et al*. 2006 emend. Wang *et al*. 2024, 6*	Emend.	[11]
*Exiguobacterium mexicanum* López-Cortés *et al*. 2006 emend. Wang *et al*. 2024, 6*	Emend.	[11]
*Gymnodinialimonas phycosphaerae* Peng *et al*. 2023 emend. Shin *et al*. 2024, 262	Emend.	[12]
*Halarchaeum solikamskense* Saralov *et al*. 2014 pro synon. *Halarchaeum nitratireducens* Minegishi *et al*. 2013	Synon.	[13]
*Haloarcula amylolytica* Yang *et al*. 2007 emend. Ma *et al*. 2024, 9*	Emend.	[14]
*Halomonadaceae* Franzmann *et al*. 1989 emend. Flores-Félix *et al*. 2025, 10*	Emend.	[5]
*Ketobacteraceae* Chuvochina *et al*. 2024 emend. Flores-Félix *et al*. 2025, 10*	Emend.	[5]
*Kitasatospora cineracea* Tajima *et al*. 2001 emend. Bouznada *et al*. 2023, 1333	Emend.	[15]
*Kitasatospora niigatensis* Tajima *et al*. 2001 pro synon. *Kitasatospora cineracea* Tajima *et al*. 2001	Synon.	[15]
*Kocuria polaris* Reddy *et al*. 2003 pro synon. *Kocuria rosea* (Flügge 1886) Stackebrandt *et al*. 1995	Synon.	[16]
*Lactococcus intestinalis* Sun *et al*. 2024 pro synon. *Lactococcus muris* Afrizal *et al*. 2023	Synon.	[17]
*Lactococcus muris* Afrizal *et al*. 2023 emend. Zhu and Gu 2025, 3*	Emend.	[17]
*Leeuwenhoekiella* Nedashkovskaya *et al*. 2005 emend. Machii *et al*. 2025, 11*	Emend.	[18]
*Limnospira fusiformis* (Woronichin 1934) Nowicka-Krawczyk *et al*. 2019 pro synon. *Limnospira maxima* (Setchell and Gardner 1917) Nowicka-Krawczyk *et al*. 2019	Synon.	[19]
*Limnospira indica* (Desikachary and Jeeji Bai 1992) Nowicka-Krawczyk *et al*. 2019 pro synon. *Limnospira maxima* (Setchell and Gardner 1917) Nowicka-Krawczyk *et al*. 2019	Synon.	[19]
*Limnospira* Nowicka-Krawczyk *et al*. 2019 emend. Pinchart *et al*. 2024, 9*	Emend.	[19]
*Listeria* Pirie 1940 (Approved Lists 1980) emend. Bouznada *et al*. 2025, 19*	Emend.	[20]
*Marinobacteraceae* Liao *et al*. 2021 emend. Flores-Félix *et al*. 2025, 10*	Emend.	[5]
*Marinomonadaceae* Chuvochina *et al*. 2024 emend. Flores-Félix *et al*. 2025, 10*	Emend.	[5]
*Methanimicrococcus* corrig. Sprenger *et al*. 2000 emend. Protasov *et al*. 2024, 12*	Emend.	[21]
*Methylobacteriaceae* Garrity *et al*. 2006 emend. Wang *et al*. 2024, 7*	Emend.	[22]
*Moraxellaceae* Rossau *et al*. 1991 emend. Flores-Félix *et al*. 2025, 10*	Emend.	[5]
*Natronospirillaceae* Kevbrin *et al*. 2020 emend. Flores-Félix *et al*. 2025, 10*	Emend.	[5]
*Neptunitalea chrysea* Yoon and Kasai 2015 emend. Sahin *et al*. 2025, 6*	Emend.	[23]
*Nocardiopsis rosea* Li *et al*. 2006 pro synon. *Nocardiopsis rhodophaea* Li *et al*. 2006	Synon.	[24]
*Nocardiopsis umidischolae* Peltola *et al*. 2002 pro synon. *Nocardiopsis tropica* Evtushenko *et al*. 2000	Synon.	[24]
*Oceanospirillaceae* Garrity *et al*. 2005 emend. Flores-Félix *et al*. 2025, 10*	Emend.	[25]
*Oceanospirillaceae* Garrity *et al*. 2005 emend. Joshi *et al*. 2025, 10*	Emend.	[5]
*Oxalobacteraceae* Garrity *et al*. 2006 emend. Flores-Félix *et al*. 2025, 10*	Emend.	[5]
*Proteinivoracaceae* corrig. Kevbrin *et al*. 2014 emend. Kevbrin and Boltyanskaya 2024, 3*	Emend.	[26]
*Pseudomonadaceae* Winslow *et al*. 1917 (Approved Lists 1980) emend. Flores-Félix *et al*. 2025, 10*	Emend.	[5]
*Pseudomonadales* Orla-Jensen 1921 (Approved Lists 1980) emend. Flores-Félix *et al*. 2025, 10*	Emend.	[5]
*Salinarimonas* Liu *et al*. 2010 emend. Messner *et al*. 2024, 9*	Emend.	[27]
*Salinisphaera halophila* Zhang *et al*. 2012 pro synon. *Salinisphaera orenii* Park *et al*. 2012	Synon.	[28]
*Salinisphaera orenii* Park *et al*. 2012 emend. Quadri *et al*. 2025, 5*	Emend.	[28]
*Salisediminibacterium haloalkalitolerans* Sultanpuram *et al*. 2015 pro synon. *Salisediminibacterium halotolerans* Jiang *et al*. 2012	Synon.	[29]
*Salisediminibacterium halotolerans* Jiang *et al*. 2012 emend. Wang *et al*. 2025, 4*	Emend.	[29]
*Schlesneria* Kulichevskaya *et al*. 2007 emend. Kulichevskaya *et al*. 2025, 9*	Emend.	[30]
*Shewanellaceae* Ivanova *et al*. 2004 emend. Flores-Félix *et al*. 2025, 10*	Emend.	[5]
*Streptomyces asiaticus* Sembiring *et al*. 2001 emend. Nouioui *et al*. 2024, 21*	Emend.	[31]
*Streptomyces violarus* (Artamonova and Krassilnikov 1960) Pridham 1970 (Approved Lists 1980) pro synon. *Streptomyces violaceus* (Rossi Doria 1891) Waksman 1953 (Approved Lists 1980)	Synon.	[32]
*Streptomyces violaceus* (Rossi Doria 1891) Waksman 1953 (Approved Lists 1980) emend. Long *et al*. 2024, 5*	Emend.	[32]
*Tenuifilum* Podosokorskaya *et al*. 2021 emend. Podosokorskaya *et al*. 2025, 6*	Emend.	[33]
*Tepidanaerobacter* Sekiguchi *et al*. 2006 emend. Westerholm *et al*. 2011, 265	Emend.	[34]
*Thermodesulfovibrio islandicus* Sonne-Hansen and Ahring 2000 pro synon. *Thermodesulfovibrio yellowstonii* Henry *et al*. 1994	Synon.	[35]
*Woeseiales* Chuvochina *et al*. 2024 emend. Lian *et al*. 2025, 8*	Emend.	[36]

* The journal in which the name was effectively published does not have continuous pages.

For references to Lists of Changes in Taxonomic Opinion no. 1-41 see *Int J Syst Evol Microbiol* **55** (2005) 7, 1403; **56** (2006) 11, 1463; **57** (2007) 4, 1376; **58** (2008) 5, 1515; **59** (2009) 5, 1559; **60** (2010) 5, 1482; **61** (2011) 4, 1504; **62** (2012) 7, 1448; **63** (2013), 8, 2371, **64** (2014), 8, 2191; **65** (2015), 7, 2028; **66** (2016), 7, 2469; **67** (2017), 7, 2081; **68** (2018), 7, 2137; **69** (2019), 13, 1850; **70** (2020), 9, 4061; **71** (2021), 004596, 004816; **72** (2022), 005164, 005413; **73** (2023), 005696, 005923; **74** (2024), 006145, 006482; **75** (2025), 006561.

## Correction to List of Changes in Taxonomic Opinion no. 40 [37]

*Haloplanus rallus* Cho *et al*. 2018 pro synon. *Haloplanus salinarum* Hwang *et al*. 2017 is given in reference [11] and not in reference [9].

## Corrections to List of Changes in Taxonomic Opinion no. 41 [38]

The synonymy proposals for *Halobellus ramosii* Pérez-Davó *et al*. 2015, *Haloferax lucentense* corrig. Gutierrez *et al*. 2004 and *Haloferax alexandrinum* corrig. Asker and Ohta 2002 were already listed in the List of Changes in Taxonomic Opinion no. 40 [37].

*Paracraurococcus* Saitoh *et al*. 1998 emend. Kingkaw *et al*. 2024 should be *Paracraurococcus* Saitoh *et al*. 1998 emend. Kingkaew *et al*. 2024. *Peptoniphilus faecalis* Ryu *et al*. 2021 has priority over *Peptoniphilus porci* Wylensek *et al*. 2021. Therefore, the comment ‘The authors proposed *Peptoniphilus porci* Wylensek *et al*. 2021 pro synon. *Peptoniphilus faecalis* Ryu *et al*. 2021. However, *P. porci* has priority, so they should have proposed *Peptoniphilus faecalis* Ryu *et al*. 2021 pro synon. *Peptoniphilus porci* Wylensek *et al*. 2021’ is incorrect.

We thank S. Turner (NCBI, NIH, Bethesda, MD, USA) for alerting us to the errors in the List of Changes in Taxonomic Opinion no. 41.

## Abbreviations

ICNP: International Code of Nomenclature of Prokaryotes
IJSEM: International Journal of Systematic and Evolutionary Microbiology
